# Asymptomatic malaria and nurturing factors in lowlands of Ethiopia: A community based cross-sectional study

**DOI:** 10.1371/journal.pgph.0000659

**Published:** 2022-08-16

**Authors:** Endale Mengesha, Meseret Dessalegne Zerefa, Habteyes Hailu Tola

**Affiliations:** 1 Water and Public Health Stream, Ethiopian Institute of Water Resources, Addis Ababa University, Addis Ababa, Ethiopia; 2 Tuberculosis/HIV Research Directorate, Ethiopian Public Health Institute, Addis Ababa, Ethiopia; University of Embu, KENYA

## Abstract

Although asymptomatic malaria cases are reservoirs of malaria parasites, there is limited evidence on the burden and nurturing factors in malaria endemic areas during dry season. Thus, this study aims to determine the prevalence of asymptomatic malaria infection and nurturing factors in endemic areas of Ethiopia during dry season.A community based cross-sectional study was conducted in malaria endemic areas in Ethiopia. Six villages with a total of 1,366 households from three malaria endemic regions of Ethiopia were selected by stratified random sampling method. One asymptomatic member of the household was randomly selected from each household. A structured questionnaire was used to collect data on socio-demographic and other factors. Finger prick blood samples for malaria rapid diagnostic test (RDT) and blood film were collected and examined. Multivariable logistic regression model was used to determine the nurturing factors with asymptomatic malaria infection. The prevalence of asymptomatic malaria infection was 7.7% with both blood film microscopic examination and malaria RDT. *Plasmodium falciparum* was the predominantly observed type of malaria species (48.0%). The presence of bodies of water around the households (adjusted odds ratio (AOR = 5.4; 95% CI (2.7 ─ 9.7); p < 0.000), infrequent indoor residual spray (IRS) applied four to six months ago (AOR = 3.5; 95% CI (1.0─11.6); p = 0.045) and more than six months (AOR = 5.2; 95% CI (1.3─20.5); p = 0.019) and personal protection measure for malaria prevention (LLIN, repellent and clothing) (AOR = 0.41; 95% CI (0.2 ─ 0.9); p = 0.028) were associated significantly with asymptomatic malaria infection. The prevalence of asymptomatic malaria infection during dry season was considerable. Strong interventions that target stagnant bodies of water, infrequent household IRS spray and personal protection measure for malaria prevention is required to decrease asymptomatic malaria infection during dry season.

## Introduction

Malaria is a vector borne disease caused by plasmodium parasites, transmitted to humans through infected female *Anopheles* mosquitoes [1]. It is a major public health problem across the world [1]. Both *Plasmodium falciparum (P*.*f)* and *Plasmodium vivax (P*.*v)* are the major malaria parasites in Ethiopia [2].

A total of 87 malaria endemic countries contributed to an estimated 229 million malaria cases globally in 2019 [3]. The majority of global malaria cases are reported from the African region due to environmental and other malaria favoring factors [3]. Ethiopia is among the highest malaria burden countries with 60% of the population living in low to high malaria risk areas, making malaria a leading public health problem in the country [2]. The incidence rate of clinical malaria was dropped from an average of 43.1 to 29.0 cases per 1000 population and deaths from 2.1 to 1.1 per 100,000 people annually for the years 2001 to 2010, and 2011 to 2016, respectively [4, 5]. As a systematic review study reported in 2021, the prevalence of malaria among adults is 13.61% [6]. A retrospective study conducted in Eastern Ethiopia, malaria elimination area showed a 69.2%, 30.6% and 0.2% incident cases of Plasmodium falciparum, P. vivax and mixed infections, respectively [5]. Moreover, a cross sectional study conducted in both stable and unstable malaria transmission areas of Ethiopia, identified 1.8% peripheral malaria parasitemia in women attending antenatal care (ANC) in unstable transmission areas, while 10.4% in stable areas [7].

There was shrinkage in the malaria transmission map and high transmission is limited mainly to the western international border area. Proportion of *P*. *falciparum* malaria remained nearly unchanged from 2000 to 2016 indicating further efforts are needed to suppress transmission [4].

According to the sub-district level malaria stratification, there was shrinkage in the malaria transmission map and about 70% of the sub-districts have achieved elimination targets.

Asymptomatic malaria infection is caused by all types of plasmodium species which favor prolonged malaria transmission in the community without clinical symptoms manifestation making diagnosis difficult [8]. Asymptomatic malaria infection is more prevalent in malaria endemic areas such as sub-Saharan Africa countries creating a stable malaria burden [9, 10]. Small scale studies indicate that the prevalence of asymptomatic malaria infection is very common in malaria endemic areas in Ethiopia [11]. A school based cross-sectional study reported from northwestern Ethiopia indicated that the prevalence of asymptomatic malaria infection is 6.8% [11]. Another study reported from south-central Ethiopia also showed that the prevalence of asymptomatic malaria infection in apparently healthy individuals is 5.0% by microscopy, while 8.2% by RDT [12]. Moreover, a cross-sectional study reported from northwest Ethiopia indicated that prevalence of asymptomatic malaria infection is 6.7% in adult participants [13]. A community-based study reported from southern Ethiopia also showed that the prevalence of asymptomatic malaria infection in pregnant women is 9.1% with microscopy and 9.7% with RDTs [14].

Several factors have been reported that are associated with asymptomatic malaria infection. For instance, age group (15–29 years), poor bed net usage, previous history of malaria/recurrent episodes of symptomatic parasitemia, co-infection with invasive bacterial disease, and cognitive impairments are factors that have a significantly associate with the prevalence of asymptomatic malaria infection [9–14]. Although the burden of asymptomatic malaria is high and well-studied in the rainy season in lowlands of Ethiopia, there is limited evidence on the burden of asymptomatic malaria infection and associated factors during the dry season. Thus, this study was aimed to determine the prevalence of asymptomatic malaria infection and nurturing factors in malaria endemic areas lowlands of Ethiopia during the dry season.

## Methods and materials

### Ethics statement

Ethical approval to conduct the study was obtained from Addis Ababa University, Institutional Review Board. Supportive letters to conduct the study were obtained from Federal Ministry of Health, Addis Ababa University and Regional Health Bureau. Permission was requested from local administration’s administrators while informed written consent was obtained from each respondents/legal guardians prior for the interview; sample drawing and respondents were participated based on their willingness. According to national malaria diagnosis and treatment guideline, the clinical malaria cases were treated immediately at the health post. Confidentiality of all information was kept; the questioners were locked in the shelf.

### Study design and setting

**Fig 1:**
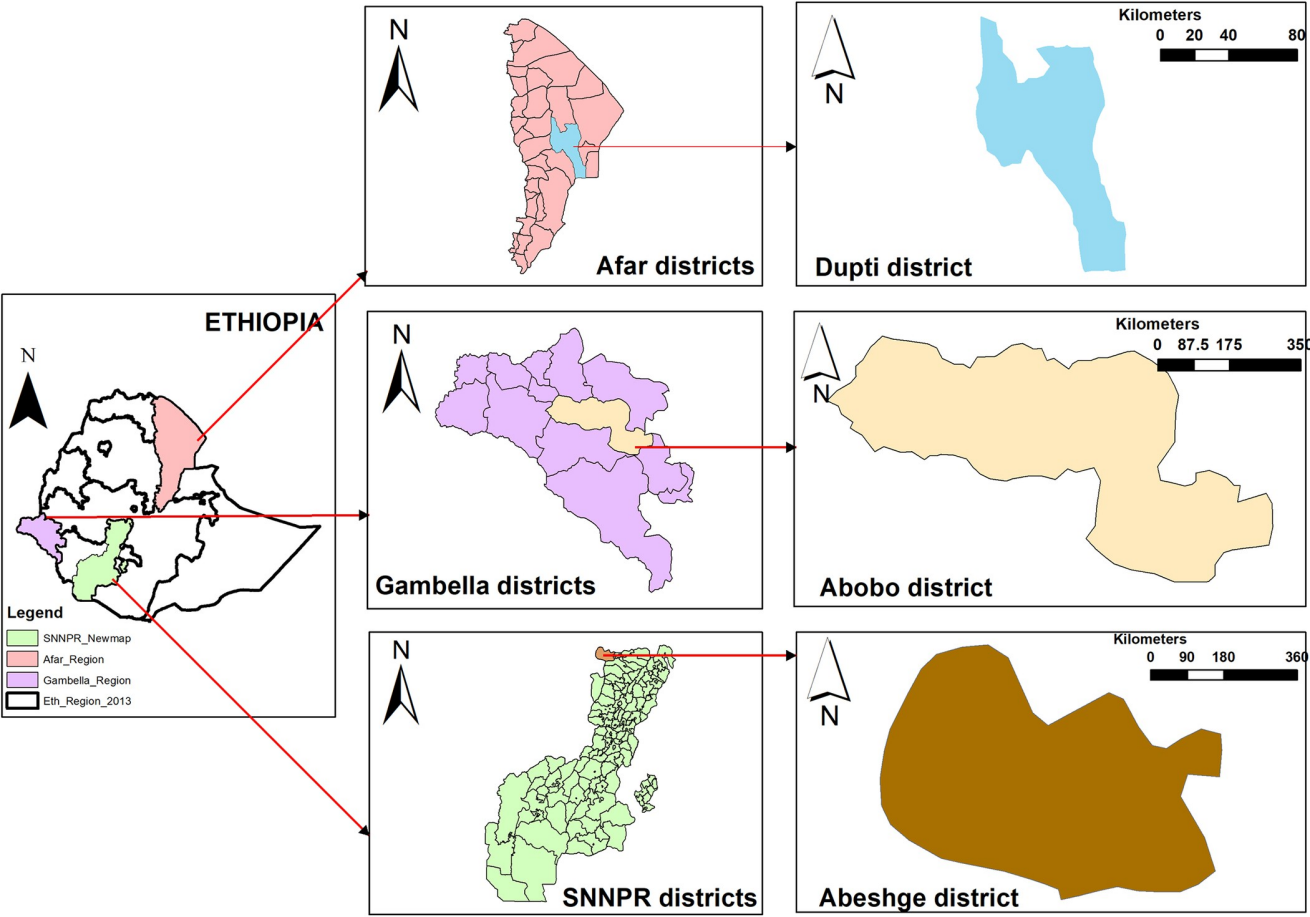
Localization of study area (shape files taken from: https://ethiopia.africageoportal.com/search?collection=Dataset).

A community-based cross-sectional study was conducted Dupti, Abobo and Abeshge districts of Afar, Gambella and Southern Nations, Nationalities, and Peoples (SNNNP) regional states of Ethiopia, respectively (Fig 1).

**Abeshge** district is located in SNNNP regional state and located southwest 167 km from Addis Ababa. Based on the recent population estimation the total population of the district was 61,424 [15]. **Dupti** district is found in Afar regional state 591 km to the north-east of Addis Ababa. The total population of the district is 65,342 inhabitants [15]. Awash River splits the district into northern and southern parts. January and February are dry seasons [16]. **Abobo** district is also found in Gambella regional state and located at southwest 815 km from Addis Ababa. The total population of Abobo district is 19,458 [15]. The district has a hot and humid climate and about 9 months of rainy season [17]. The dry months of the district are January and February [17]. There is a manmade dam on Alwero river in the district which could facilitate malaria transmission.

### Sampling technique and sample size

The regions and districts were selected purposively based on their malaria burden in the country. Two villages were selected from each district by a stratified random sampling method. Households were also selected by systematic random sampling by using health post household registration book as a sampling frame. From each household one asymptomatic member was randomly selected and enrolled into this study.

Ninety percent of the true population proportion [12], 95% confidence level, 3% margin of error, 5% non-response rate and two design effect were considered in sample size calculation. Accordingly, a total of 1,390 households were determined using single population proportion. Six villages from three districts of three malaria endemic regions of Ethiopia were selected by stratified random sampling method. The households were proportionally allocated to each selected village population size and selected by systematic random sampling technique. One asymptomatic member of the household was selected by simple random sampling from each selected household.

### Inclusion and exclusion criteria

All individuals without signs and symptoms of malaria were included into this study. However, individuals with febrile illness, severely sick, and mentally incapable were excluded from this study.

### Data collection tools and procedure

Literature based structured questions were used for data collection on socio-demographic and other important variables. The English questions were translated into local languages (Amharic and Afaan Oromo). Laboratory test supplies used to RDT and microscopic examination (RDT kits and supplies such as sterile lancets, alcohol swabs and frosted and non-frosted microscope slides) were used for laboratory data collection. Training was provided for two days on the objectives of the study, data collection tools and methods, specimen collection techniques, informed consent and ethical issues for data collectors and project staff. The data collectors interviewed the study participants in their home after written informed consent was obtained. The laboratory professional assigned in each group collected finger prick blood samples for RDT, and blood film (thick and thin blood films) for microscopic examination immediately after the interview. On average one hour and 30 minutes was taken per household for both interview and specimen collection. The participants who were RDT positive were treated with artemisinin-based combination therapy (ACT) as per the national guideline at health post level or referred to the nearby health center or hospitals for further diagnosis and management.

All data collectors were supervised twice a day for data quality by the assigned study supervisor. The supervisor randomly checked the filled questionnaire for completeness and consistency. At the end of the day all the checked questionnaires and blood film slides were collected and sealed in a bag provided by the supervisors while the RDT was disposed at the health post as per safety procedures.

### Data analysis

Data were entered into Microsoft Excel 2013 sheet and exported into IBM statistical package for social sciences (SPSS) version 20.0 for the analysis. The filled questionnaires were properly coded and cross checked with original data to ensure validity before the main data analysis. Laboratory test results by both blood film microscopic examination and RDT were treated as an outcome variable, and it was categorized into “positive” and “negative” to explore the associations with possible risk factors of asymptomatic malaria infection. Descriptive statistics such as frequency and percent were calculated to show the participants distribution of asymptomatic malaria infection. Chi-squared and Fisher’s exact tests were conducted to determine factors associated with asymptomatic malaria infection. A multiple logistic regression model was used to determine the independent effect of factors that were associated with asymptomatic malaria infection in bivariate analysis. Variables scored ≤ 0.2 p-value at bivariate analysis were included to multivariate logistic regression by stepwise model building method. Statistical significance was set at p-value ≤ 0.05.

## Results

### Socio-demographic characteristics of participants

A total of 1,366 participants were enrolled in this study with 98.2% response rate. Of the total participants, 958 (70.1%) were rural residents and 689 (50.4%) were residents around bodies of water. About 39.0% of the participants were illiterate, while 40.6% attended elementary school (Table 1). Fifty four percent of the participants were female and 51% were in the age range of 15 to 44 year (Table 1).

**Table 1:** Socio-demographic characteristics of sampled population.

Variables		Frequency	Percent
Residence	Urban	408	29.9
	Rural	958	70.1
Age	<5	77	5.6
	5–14	412	30.2
	15–44	696	51.0
	45–64	114	8.3
	>64	67	4.9
Sex	Female	738	54.0
	Male	628	46.0
Educational Status	High school and above	278	20.4
	Elementary	555	40.6
	Illiterate	533	39.0
Occupational status	Government employee	81	5.9
	Self-employee	203	14.9
	Student	465	34.0
	Housewife	385	28.2
	Farmer	232	17.0
Availability of water bodies	No	677	49.6
	Yes	689	50.4
Type of water bodies	Not Applicable (No Water Body)	677	49.6
	Perennial Surface Water	370	27.1
	Ephemeral Surface Water	45	3.3
	Man Made Surface Water	274	20.1
Availability of bed nets	Yes	1280	93.7
	No	86	6.3
Utilization of Bed nets	Yes	1281	93.8
	No	85	6.2
General Condition of the net	Good (no holes)	902	66.0
	Fair (no holes that fit a torch battery)	205	15.0
	Poor (1 or more holes that fit a torch battery)	259	19.0
The time when Indoor residual spray was applied	≤ 3 months	127	9.3
	4–6 months ago,	1021	74.7
	>6 months ago,	218	16.0
Malaria prevention measure applied	Chemoprophylaxis	369	27.0
	Personal Protection (LLIN, repellent etc.)	253	18.5
	Mosquito control (IRS, draining etc.)	313	22.9
	Both Personal protection and Environmental control not applied	431	31.6
Participants who received health education	Yes	1021	74.7
	No	345	25.3

### The prevalence of asymptomatic malaria infection

A total of 1,366 microscopy diagnosis on thick and thin blood films and an equivalent number of malarias RDTs were examined for asymptomatic malaria infection. The prevalence of asymptomatic malaria infection was 8.1% (n = 111) with microscopy, while 9.3% (n = 127) with RDT. The overall prevalence of asymptomatic malaria infection by both blood film microscopy examination and RDT was 7.7% (n = 105; 95% CI: (5.2–8.7)). Of the prevalent cases, 48.0% was *P*.*f*, while 27.9% *P*.*V* and 23.4% both *P*.*f* and *P*.*V*.

### Bivariate analysis

Table 2 depicts the variables associated with asymptomatic malaria infection by bivariate analysis. Rural residence (unadjusted odds ratio (UOR) = 2.0; 95% CI (1.2─3.4); p = 0.007), availability of water (UOR = 4.1; 95% CI (2.5─6.6); p = 0.000), perennial surface water (UOR = 3.3; 95% CI (1.9─5.7); p < 0.000), ephemeral surface water (UOR = 7.4; 95% CI (3.2─17.3); p < 0.000), manmade surface water (UOR = 4.6; 95% CI (2.7─8.0); p < 0.000), no application for both personal protection and mosquito control measure for malaria prevention (UOR = 2.0; 95% CI (1.2─3.3); p = 0.010) and absence of health education (UOR = 1.7; 95% CI (1.0─2.5); p = 0.049) were significantly associated with asymptomatic malaria infection.

**Table 2:** Bivariate analysis of nurturing factors associated with asymptomatic malaria infection.

Variables		Negative, n (%)		Positive, n (%)		UOR (95%CI)	P-Value
Residence	Urban	389	95	19	4.7	1	
	Rural	872	91	86	9.0	2.0 (1.2─3.4)	0.007
Age	<5	68	88	9	11.7	0.6 (0.3─1.4)	0.258
	5–14	380	92	32	7.8	0.6 (0.2─1.1)	0.146
	15–44	647	93	49	7.0	0.7 (0.2─1.4)	0.51
	45–64	104	91	10	8.8	0.6 (0.2─1.9)	0.397
	>64	62	93	5	7.5	1	
Sex	Female	686	93	52	7.0	1	
	Male	575	92	53	8.4	1.2 (0.8─1.8)	0.336
Educational Status	High school and above	257	92	21	7.6	1	
	Elementary	510	92	45	8.1	1.1 (0.6─1.8)	0.78
	Illiterate	494	93	39	7.3	0.9 (0.6─1.7)	0.903
Occupational status	Government employee	74	91	7	8.6	1	
	Self-employee	190	94	13	6.4	0.7 (0.3─1.9)	0.507
	Student	429	92	36	7.7	0.9 (0.4─2.1)	0.781
	Housewife	362	94	23	6.0	0.7 (0.3─1.6)	0.377
	Farmer	206	89	26	11.2	1.3 (0.6─3.2)	0.519
Availability of water bodies	No	655	97	22	3.2	1	
	Yes	606	88	83	12.0	4.1 (2.5─6.6)	0.000
Type of water bodies	Not Applicable (No Water Body)	655	97	22	3.2	1	
	Perennial Surface Water	333	90	37	10.0	3.3 (1.9─5.7)	0.000
	Ephemeral Surface Water	36	80	9	20.0	7.4 (3.2─17.3)	0.000
	Man Made Surface Water	237	87	37	13.5	4.6 (2.7─8.0)	0.000
Availability of bed nets	Yes	1181	92	99	7.7	1	
	No	80	93	6	7.0	0.9 (0.4─2.1)	0.799
Utilization of Bed nets	Yes	1185	93	96	7.5	1.5 (0.7─3.0)	0.302
	No	76	89	9	10.6	1	
General Condition of the net	Good (no holes)	823	91	79	8.8	1	
	Fair (no holes that fit a torch battery)	194	95	11	5.4	0.6 (0.31─1.1)	0.112
	Poor (1 or more holes that fit a torch battery)	244	94	15	5.8	0.6 (0.4─1.1)	0.126
The time when Indoor residual spray was applied	≤ 3 months	124	98	3	2.4	1	
	4–6 months ago,	932	91	9	8.7	3.9 (1.2─12.7)	0.021
	>6 months ago,	205	94	13	6.0	2.6 (0.7─9.4)	0.139
Malaria prevention measure applied	Chemoprophylaxis	346	94	23	6.2	1	
	Personal Protection (LLIN, repellent etc.)	242	96	11	4.3	0.7 (0.3─1.4)	0.312
	Mosquito control (IRS, draining etc.)	292	93	21	6.7	1.1 (0.6─2.0)	0.801
	Both Personal protection and Environmental control not applied	381	88	50	11.6	2.0 (1.2─3.3)	0.010
Participants who received health education	Yes	934	92	87	8.5	1	
	No	327	95	18	5.2	1.7 (1.0─2.5)	0.049

### Multivariate analysis

In multivariable analysis, presence of bodies of water around the households (adjusted odds ratio (AOR = 5.4; 95% CI (2.7─9.7); p < 0.000), indoor residual spray (IRS) applied four to six months ago (AOR = 3.5; 95% CI (1.0─11.6); p = 0.045) and above six month ago (AOR = 5.2; 95% CI (1.3─20.5); p = 0.019) were increased the risk of asymptomatic malaria infection significantly [Table 3]. However, personal protection measures for malaria prevention (LLIN, repellent and clothing) (AOR = 0.41; 95% CI (0.2─0.9); p = 0.028) was decreased the risk of asymptomatic malaria infection significantly (Table 3). Residence, type of body of water, and health education lost their significance after adjusting for the confounder.

**Table 3:** Multivariable analysis of nurturing factors associated with asymptomatic malaria infection.

Variable		Negative for Malaria Parasite n (%)		Positive for Malaria Parasite n (%)		AOR (95%CI)	P. Value
Availability of water bodies	No	655	97	22	3.2	1	
	Yes	606	88	83	12	5.4 (2.7─9.7)	0.000
The time when Indoor residual spray was applied	≤ 3 months	124	98	3	2.4	1	
	4–6 months ago,	932	91	89	8.7	3.5 (1.0─11.6)	0.045
	>6 months ago,	20	94	13	6	5.2 (1.3─20.5)	0.017
Malaria prevention measure applied	Chemoprophylaxis	346	94	23	6.2	1	
	Personal Protection (LLIN, repellent etc.)	242	96	11	4.3	0.41(0.2 ─ 0.9)	0.028
	Mosquito control (IRS, draining etc.)	292	93	21	6.7	0.65 (0.3─1.3)	0.211
	Both Personal protection and Environmental control not applied	381	(88.4	50	11.6	0.95 (0.5─1.8)	0.858

## Discussion

Mosquito population fluctuates with seasonal patterns or presence of bodies of water which leads to unstable transmission in the area [18]. Presence of asymptomatic malaria cases are critical for mosquito infection because they are the reservoir of the gametocyte stage of the parasites which facilitates prolonged transmission [19]. Although the problem related to asymptomatic malaria cases is well known, no evidence is generated on the burden and the nurturing factors associated with asymptomatic malaria during the dry season in in the study area. Thus, this study was aimed to determine the prevalence and nurturing factors of asymptomatic malaria during the dry season in three lowland districts of Ethiopia. In the current study the overall prevalence of asymptomatic malaria by both microscopy and RDT was 7.7% (microscopy = 8.1% and RDT = 9.3%). This finding is relatively high compared with studies conducted in northwest (6.8% overall) and south-central Ethiopia [12–14]. This difference is probably due to geographical location, sampling methods and seasonal variations. In addition, the difference between the present study result and the previous study could be due to the presence of large bodies of water (Alwero dam, Awash and Gibe rivers) in the current study areas which could create a favorable environment for malaria transmission and lead to high prevalence of asymptomatic malaria. The presence of irrigation from these bodies of water could be also the main reason of the difference between the present study result and previously reported study findings.

Presence of bodies of water around the households, household sprayed four to six months ago, and above six months, personal protection measures for malaria prevention (LLIN, repellent and clothing) and being a rural resident were significantly associated with the prevalence of asymptomatic malaria infection.

In the current study, there was significant association between presence of bodies of water around the households of study participants and the prevalence of asymptomatic malaria infection. This finding is similar with the study reported from northern Ethiopia in which individuals who live near stagnant water were at higher risk of acquiring malaria during the dry season [13].

In the current study, a 59% decrease in odds of asymptomatic malaria infection with the use of personal protection measure (LLIN, repellent etc.) were reported. This result is similar with the previous study finding in which individuals who lacks LLIN were at higher risk of asymptomatic malaria infection compared with those who owned and used personal protective measure [13, 20]. Moreover, the study reported from Cambodia indicated that in-house protective measures significantly decreased the odds of asymptomatic malaria infection [21]. This finding is similar with the present study findings.

Households not sprayed or sprayed four and above months ago with IRS showed a significant association with the prevalence of asymptomatic malaria infection in the current study. This finding was like the findings of the study reported from northern Uganda and Zambia in which regular and careful management of IRS significantly reduce malaria infection [22, 23]. Furthermore, the study from eastern Ethiopia reported similar finding with the present study findings in which malaria prevalence was decreased following a regular spraying of IRS [24].

Age, occupation, educational status, and bed net usage were not significantly associated with asymptomatic malaria infection in the current study. This finding was similar to the results of the study reported from Eritrea which indicated the absence of a significant association between asymptomatic malaria infection and participants sex, age, education status, occupation, bed net usage [25]. However, a study from Sanja town from northwest Ethiopia reported findings contradicting our result in which a significant association was reported between asymptomatic malaria infection and age, education status, occupation, and bed net usage [11]. This difference might be due to the study population and season in which the study was conducted. In the case of a study reported from northern Ethiopia, the study population were school children, whereas in our study the study population was the general population [11].

The findings of the present study provide a clear message for malaria control program efforts. Although the burden of malaria is decreasing across the world, the prevalence of asymptomatic malaria infection during dry season in the current study is considerable and needs to be addressed by the malaria program. The presence of bodies of water is one of the main predictors of asymptomatic malaria infection during dry season. Thus, spraying bodies of water during the dry season could decrease the burden of asymptomatic malaria infection. Moreover, our result indicates that special attention should be paid to frequent spray of households before 6 months to decrease asymptomatic malaria infection burden.

The main limitation of this study was recall bias on the use of malaria prevention measure and history of previous malaria infection which may affect the nurturing factors identification. In addition, targeting only one season was another limitation of this study that could limit the generalizability of this study findings. Therefore, in future studies of asymptomatic malaria burden and the risk factors during all seasons by addressing history of previous episodes will be important to increase the generalizability of the results.

## Conclusion

The overall prevalence of asymptomatic malaria infection in malaria endemic regions of Ethiopia during dry season is considerable. All policies and strategies designed for malaria elimination in Ethiopia must address the significant number of asymptomatic malaria carriage to interrupt the transmission cycle of symptomatic malaria. In addition to this, strong interventions targeting bodies of water, in the surrounding and personal protectives means need to be applied.

## Supporting information

S1 Data Datasets analyzed for the finding of “Asymptomatic malaria and nurturing factor”. (ZIP)

S2 Data Saved base layer map on the ArcMap used to generate the figure for study area localization before exporting the map. (MXD)
